# Pulmonary sclerosing pneumocytoma in an 18-year-old male patient: A case report and literature review

**DOI:** 10.1097/MD.0000000000020869

**Published:** 2020-06-26

**Authors:** Huu Y Le, Dinh Phuc Pham, Khac Tuyen Nguyen, Van Ai Hoang, The Son Trinh, Quyet Do

**Affiliations:** a Center of Respiratory Diseases, 103 Military Hospital; b Center of Oncology; c Department of Pathology, 103 Military Hospital; d Military institute of clinical embryology and histology, Vietnam Military Medical University, Hanoi, Vietnam; e Director of Vietnam Military Medical University, Hanoi, Vietnam.

**Keywords:** computed tomography scan, pulmonary sclerosing pneumocytoma, thyroid transcription factor-1

## Abstract

**Rationale:**

Pulmonary sclerosing pneumocytoma (PSP) is a rare benign tumor of the lung, mostly presented in Asian middle-aged women. Initially, it was considered as a vascular origin tumor, but then research evidence showed that it was derived from natural epithelial tissue. On imaging, this tumor may be found as a solitary well-circumscribed lung parenchymal lesion, and is often located in juxtapleural or juxtafissural positions. On histopathology, it consists of cuboidal surface cells and stromal round cells, both of which are positive for thyroid transcription factor-1. Here we report a case of a young PSP male patient and review the relevant literature in order to improve our understanding of this disease.

**Patient concerns:**

An 18-year-old man was referred to our hospital after accidentally finding a lesion on chest X-ray. Contrast-enhanced computed tomography showed a soft tissue mass with homogeneous enhancement in the left lower lobe posterior segment.

**Diagnoses:**

The diagnosis of PSPs was confirmed by histopathological examination.

**Interventions and outcomes:**

The patient underwent a thoracoscopic wedge resection and was followed-up after that. One month later, he had good performance status with no recurrent tumors.

**Lessons:**

PSP in a young man is really uncommon, and is confused with malignant tumors. A histopathological examination is considered as the diagnostic gold standard for this uncommon tumor. Surgery is the main treatment.

## Abstract 2

Supplemental Digital Content is available in the text

## 1 Introduction

Pulmonary sclerosing pneumocytoma (PSP), which was first described as pulmonary sclerosing hemangioma by Liebow et al, in 1956,^[1]^ is a rare benign neoplasm. It mostly occurs in adults over 50 years old and the incidence in women is 5 times higher than in men.^[2,3]^ This tumor was initially thought to be vascular in origin. However, it is currently considered as epithelial in nature and was named PSP according to the 2015 World Health Organization Classification of lung tumors.^[3,4]^

On imaging, PSP is a solitary well-circumscribed lung parenchymal lesion, often juxtapleural or juxtafissural in location.^[5,6]^ It often shows homogeneous enhancement.^[7,8]^ These imaging characteristics are not specific, as a result, radiology alone is not an ideal method for the definitive diagnosis.^[9]^

On histopathology, PSP contains 2 types of cells, cuboidal surface cells and stromal round cells, both of which are regarded as neoplastic.^[4,10]^ In immunohistochemistry, they are positive for thyroid transcription factor-1 (TTF-1).^[11,12]^

## 2 Case presentation

**Figure 1:**
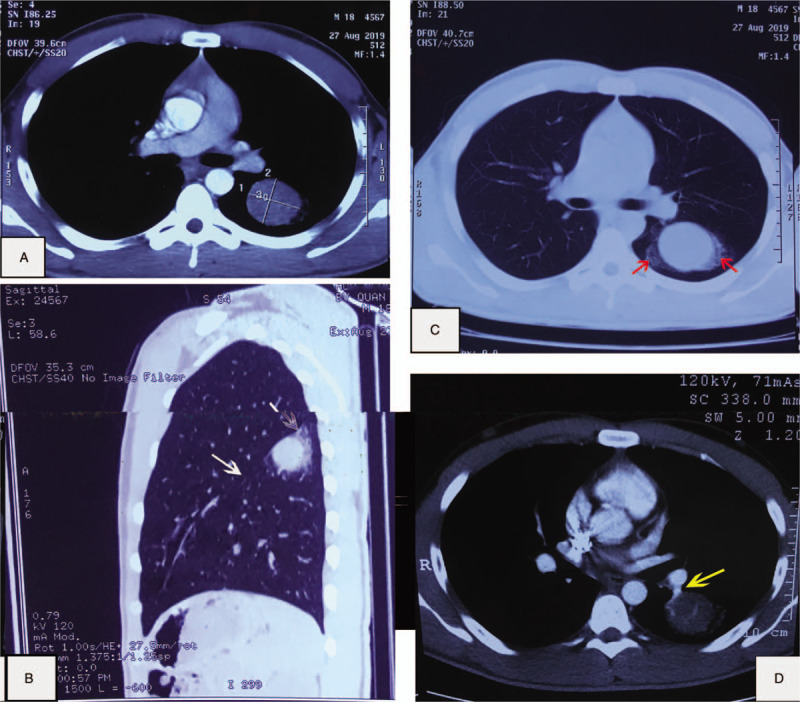
(A and B) A round lesion located in the lower left lobe in thorax CT, below the left greater fissure (white arrows). (C) A mass with surrounding ground-glass opacity (red arrows), defined as the “halo sign.” (D) CT performed 1 mo later revealed that the size had not changed. An obviously enhanced, engorged vascular structure (a yellow arrow) adjacent to the lesion. It was defined as the “overlying vessel sign.” CT = computed tomography.

**Figure 2:**
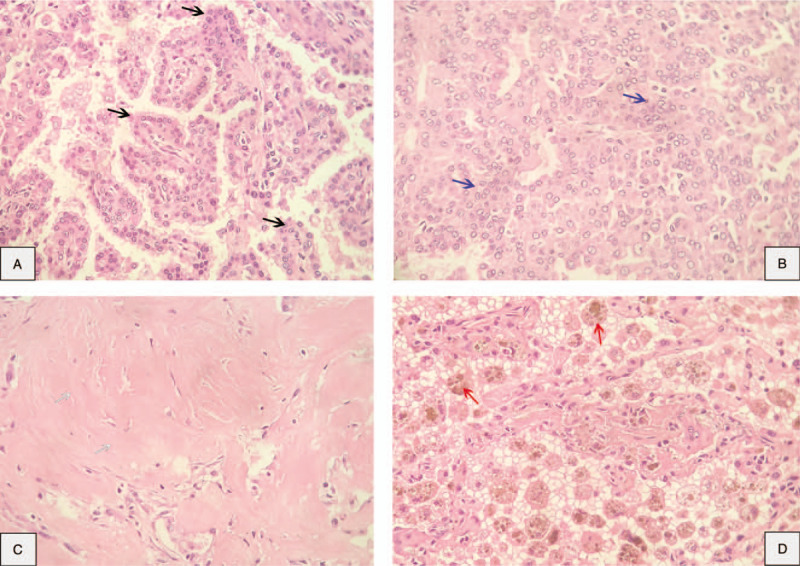
Pathological findings: pulmonary sclerosing pneumocytoma in a core-needle biopsy (hematoxylin-eosin, 40×). Two types of cells, cuboidal surface cells and stromal round cells, were organized into 4 structural patterns. (A) Papillary (black arrows). (B) Solid (blue arrows). (C) Sclerotic (white arrows). (D) Hemorrhagic (red arrows).

An 18-year-old male patient was referred to our hospital in August 2019 after accidentally detecting a solitary round lesion on the lower left lobe on chest X-ray and computed tomography (CT) scan (see Fig. 2, Supplemental Content, which demonstrates the lesion detected on chest X-ray). His medical history was normal. The patient had no symptoms of respiratory disorder, no smoking, and no tuberculosis history (see Fig. 1, Supplemental Content, which demonstrates the patient's status on admission). On admission, his vital signs and respiratory examination were normal. Laboratory tests showed that white blood cells: 6360/mm^3^, red blood cells: 4,930,000/mm^3^, hemoglobin: 15.1 g/dL, platelets: 209,000/mm^3^, urea: 30.8 mg/dL, and creatinine: 1.2 mg/dL. Two tumor markers of carcinoembryonic antigen and Cyfra 21-1 were <1.73 and 4.04 ng/mL, respectively. Contrast-enhanced thorax CT showed a soft-tissue lesion in the size of 37 × 30 mm located in the lower left lobe (below the left greater fissure), which was homogeneous and showed strong enhancement with a ground-glass opacity lesion around (Fig. 1).

**Figure 3:**
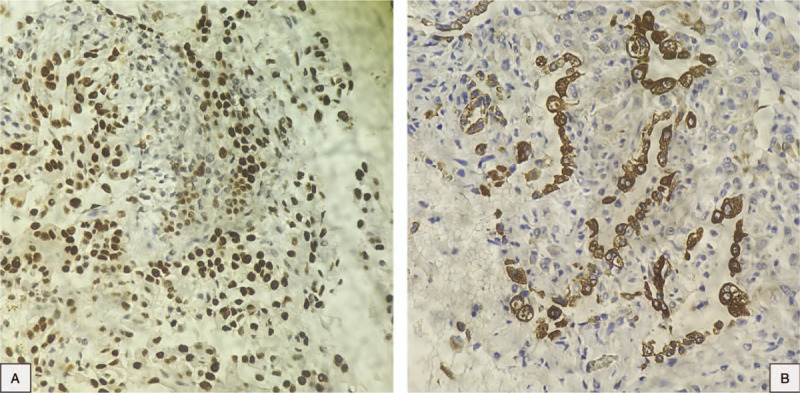
Immunohistochemical staining. (A and B) The surface cells are positive for both TTF-1 and cytokeratin AE1/AE3; the round cells are positive for TTF-1, negative for cytokeratin AE1/AE3. TTF-1 = thyroid transcription factor-1.

One month later, he underwent enhanced dynamic CT with a 16-multidetector CT scan. The results showed that the size of this tumor had not changed over time. Mean baseline tumor attenuation was 27.8 ± 8.0 Hounsfield unit (HU) (range, 25–34 HU). The mean tumor peak enhancement value was 69.9 ± 8.0 HU (range, 55–90 HU), and the mean net enhancement value was 42.1 HU. The time to peak enhancement was 60 seconds (see Fig. 3, Supplemental Content, which demonstrates the enhanced dynamic 16-multidetector CT scan with 20 seconds time interval). This tumor was initially considered as a malignant lesion which could be lung cancer or primary pulmonary lymphoma. He was counseled and agreed to be performed bronchoscopy and transthoracic biopsy. The results of bronchoscopy were clear airway and normal bronchial mucosa in both sides. Bronchoalveolar lavage fluid tests for common bacteria, tuberculosis, and fungi were all negative. The patient was diagnosed by CT-guided core-needle biopsy. A histopathological examination on formalin-fixed paraffin-embedded tissue revealed that 2 cellular components of this tumor consisted of surface epithelial cells similar to type II alveolar pneumocytes and round stromal cells, which were organized into 4 main histological patterns: papillary, sclerosing, solid, and hemorrhagic (Fig. 2). Immunohistochemical staining detected both TTF-1 and cytokeratin AE1/AE3 in the surface epithelial cells. TTF-1 was detected in the round stromal cells, but cytokeratin AE1/AE3 was not (Fig. 3). The tests for the other markers were performed. While only the round cells were positive (40%) for progesterone receptor, both types of cells were negative for estrogen receptor, weakly positive (<1%) for Ki67, and strongly positive for vimentin (see Fig. 4, Supplemental Content, which demonstrates the results in immunohistochemical staining for progesterone receptor, estrogen receptor, Ki67, and Vimentin).

This case was reviewed at the multidisciplinary tumor board of our hospital. There was an opinion that the patient should be followed-up periodically on chest X-ray instead of having surgery because of the high risk of bleeding complications. However, his large tumor raised the risk of compressing adjacent structures, we concluded that he should be performed the segmentectomy. The patient underwent a wedge resection of the posteromedial basal segment of the left lung without any complications (see Fig. 5, Supplemental Content, which demonstrates the postoperative tumor). Postoperative histopathological examinations were consistent with the previous biopsy results. After the surgery, he was followed-up, and 1 month later, no recurrent lesions were discovered on chest X-ray (see Fig. 6, Supplemental Content, which demonstrates the 1-month-postoperative chest X-ray image). The patient provided a written informed consent for this publication.

## 3 Discussion

At first, this case was difficult to make a preliminary diagnosis because previous researches had shown that PSP is mainly presented in middle-aged women, and a female-to-male ratio is from 5:1 to 7.7:1 in different populations.^[2,5,13]^ Several reports showed this ratio might be significantly higher.^[14,15]^ PSP is mostly asymptomatic, and ranges from 92.1% to 96.6% of all cases. It is accidentally detected by routine check-ups or follow-up examination of current respiratory diseases.^[2,5,13–15]^ However, Hu et al and Chen et al reported that the percentage of patients having at least 1 symptom (hemoptysis, coughing, sputum, chest pain, or fever) was 63% (29/46), 65.4% (17/26), respectively.^[16,17]^ The minor difference among these reports might be due to both racial differences and small sample sizes. According to a report by Shin et al, most patients had a single lesion (92.1%), smooth boundary (65.8%), and oval shape (65.8%) and the mean diameter was 22.7 mm. The CT signs included marginal pseudocapsule (50%), overlying vessel (26.3%), air gap (2.6%), and halo sign (17.1%). Only 4 patients (5.3%) had 2 lesions (5.3%), and 2 patients (2.6%) had >3 lesions.^[5]^ Similarly, the proportion of the solitary lesion ranges from 96.0% to 100% in several articles. The tumor is commonly located in the left lower lobe, and the presence of this tumor in the other lobes varies from study to study.^[2,13–15]^ Our case is consistent with reports of the number of lesion, the tumor location, and the CT sign. In addition, some tumors have been situated in the fissure between 2 lobes of the lung, the mediastinum, the hilum, and unknown locations ^[2,14,16]^ (See Table 1). Some studies reported that PSP could be bilateral, which should be distinguished from metastatic lung tumors.^[17–19]^

**Table 1:**
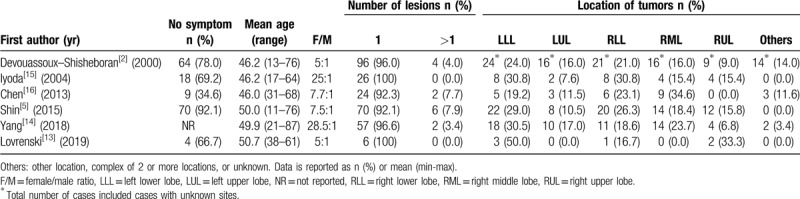
Review of demographic characteristics and clinical information of pulmonary sclerosing pneumocytoma.

The characteristics of PSP in thoracic CT scan has been described as a benign tumor with strong enhancement after intravenous administration of contrast medium,^[6]^ homogeneous enhancement with maximum CT values ranging from 90 to 110 HU.^[8]^ In this case, the mean tumor peak enhancement value was 69.9 ± 8.0 HU (range, 55–90 HU), the mean net enhancement value was 42.1 HU, and the time to peak enhancement was 60 seconds. According to a study by Yi et al, malignant nodules had higher mean peak enhancement values, mean net enhancement values, and lower mean time to peak enhancement than benign nodules. In addition, when 30 HU or more of net enhancement was set as a cutoff value to distinguish between malignant and benign nodules, the sensitivity, specificity, positive predictive value, negative predictive value, and accuracy were 99%, 54%, 71%, 97%, 78%, respectively.^[20]^ These clues led us to initially think about the stage cT2N0Mx lung cancer even though he was very young. Nevertheless, in a study by Chung et al, PSP had even more rapid and stronger enhancement than malignant nodules.^[7]^ These characteristics integrated with morphologic CT findings (ie, round or ovoid shape, smooth margin, and homogeneous attenuation) could allow differentiation between PSP and malignant nodules.

In the current World Health Organization classification of lung tumors from 2015, PSP has been changed from the “Miscellaneous tumors” group, where was previously classified in both the 1999 and 2004 versions, to the “Adenomas” group.^[4,21,22]^ In terms of histopathology, there are 4 possible histological components: papillary, sclerotic, solid, and hemorrhagic.^[15,18]^ A combination of 4 patterns is mostly observed, ranging from 50.0% to 76.9%, a 3-pattern combination followed with 19.2% to 43.8%, and a relatively small percentage of 2-pattern combination. In some literature, no single-pattern tumors have been reported. Observation results also indicated that the proportion of each pattern differs from one study to another^[2,15,16,23]^ (See Table 2). PSP includes a dual population of surface cells similar to type II pneumocytes and round cells, with marginally different histogenetic profiles.^[4]^ Immunohistochemically, the surface cells are positive for both TTF-1 and cytokeratin AE1/AE3, whereas the round cells are only positive for TTF-1. PSP has been recognized as not the tumor of vascular origin for many years. It is believed that origin of this tumor is primitive respiratory epithelium that express TTF-1.^[2,7,13,24]^ The process of this tumor diagnosis can be critically difficult in the frozen section, small biopsies, and cytology because there is the possibility of being confused with adenocarcinoma and carcinoid tumors.^[4,14,16]^

**Table 2:**
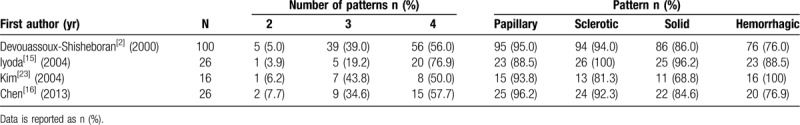
Review of histological characteristics of pulmonary sclerosing pneumocytoma.

The recurrence rate is negligible, according to Devouassoux-Shisheboran et al, Iyoda et al, Shin et al, and Hu et al, it was 0% (0/30), 3.8% (1/26), 1.3% (1/76), 0% (0/45), respectively, with the follow-up periods ranged from 1 to 228 months.^[2,5,15,17]^ Furthermore, there have been no reports showing that patients died or had any severe complications of the recurrence after surgical treatment.^[16,25]^ Several articles reported that pleural and lymph node metastases occurred in a very small percentage of PSP, but these did not negatively affect prognosis; therefore, it was thought to be benign.^[2,26,27]^ Xu et al suggested that surgical resection was curative for this tumor, and therefore no additional treatment needed after the surgery.^[27]^ In our case, CT showed there were no pleural or hilar lymph node metastases. No recurrences have appeared during follow-up by the last contact and chest X-ray review. Surgery is the basic treatment, including limited resection (enucleation or wedge) and lobectomy.^[2,13,15,17]^ Among these techniques, video-assisted thoracoscopic surgery is the most frequent procedure, in particular video-assisted thoracoscopic surgery lobectomy (50.0%).^[16]^ Additionally, some patients definitively diagnosed with PSP by biopsy were just followed through changes on chest X-ray or cured by radiotherapy for unresectable tumors.^[28,29]^

## 4 Conclusions

PSP is a rare benign lung neoplasm that typically affects Asian middle-aged women. The definitive diagnosis requires a histopathological examination with a corresponding immunohistochemical analysis. Also, it is easily mistaken for adenocarcinoma and carcinoid tumors. Surgery is still the best treatment for this disease.

## Author contributions

**Formal analysis:** Huu Y Le.

**Investigation:** Huu Y Le, Khac Tuyen Nguyen, Van Ai Hoang.

**Methodology:** Dinh Phuc Pham, Khac Tuyen Nguyen.

**Resources:** Huu Y Le, Dinh Phuc Pham, The Son Trinh.

**Supervision:** Quyet Do.

**Writing – original draft:** Huu Y Le, Van Ai Hoang.

**Writing – review & editing:** Dinh Phuc Pham, Khac Tuyen Nguyen, The Son Trinh, Quyet Do.

## Supplementary Material

Supplemental Digital Content

## Supplementary Material

Supplemental Digital Content

## Supplementary Material

Supplemental Digital Content

## Supplementary Material

Supplemental Digital Content

## Supplementary Material

Supplemental Digital Content

## Supplementary Material

Supplemental Digital Content
