# Supplementary material for: Pulmonary sclerosing pneumocytoma in an 18-year-old male patient: A case report and literature review
Source: Medicine (Baltimore). 2020 Jun 26;99(26):e20869. doi: 10.1097/MD.0000000000020869 (PMC7329001; doi:10.1097/MD.0000000000020869)
Supplement: Supplemental Digital Content [file medi-99-e20869-s003.doc]

**Pulmonary sclerosing pneumocytoma in an 18-year-old male patient: a case report and literature review**

Y Huu Le MD^a^; Phuc Dinh Pham MD^b^; Tuyen Khac Nguyen MD^c^; Ai Van Hoang MD^d^; Son The Trinh MD, PhD^d^; Quyet Do MD, PhD^d*^

^a^ Center of Respiratory Diseases, 103 Military Hospital, Hanoi, Vietnam

^b^ Center of Oncology, 103 Military Hospital, Hanoi, Vietnam

^c^ Department of Pathology, 103 Military Hospital, Hanoi, Vietnam

^d^ Vietnam Military Medical University, Hanoi, Vietnam

* Corresponding author. Tel: +84 98 330 1839, e-mail: [quyetd@vmmu.edu.vn](mailto:quyetd@vmmu.edu.vn)

*The enhanced dynamic 16-multidetector CT scan with 20 seconds time interval*


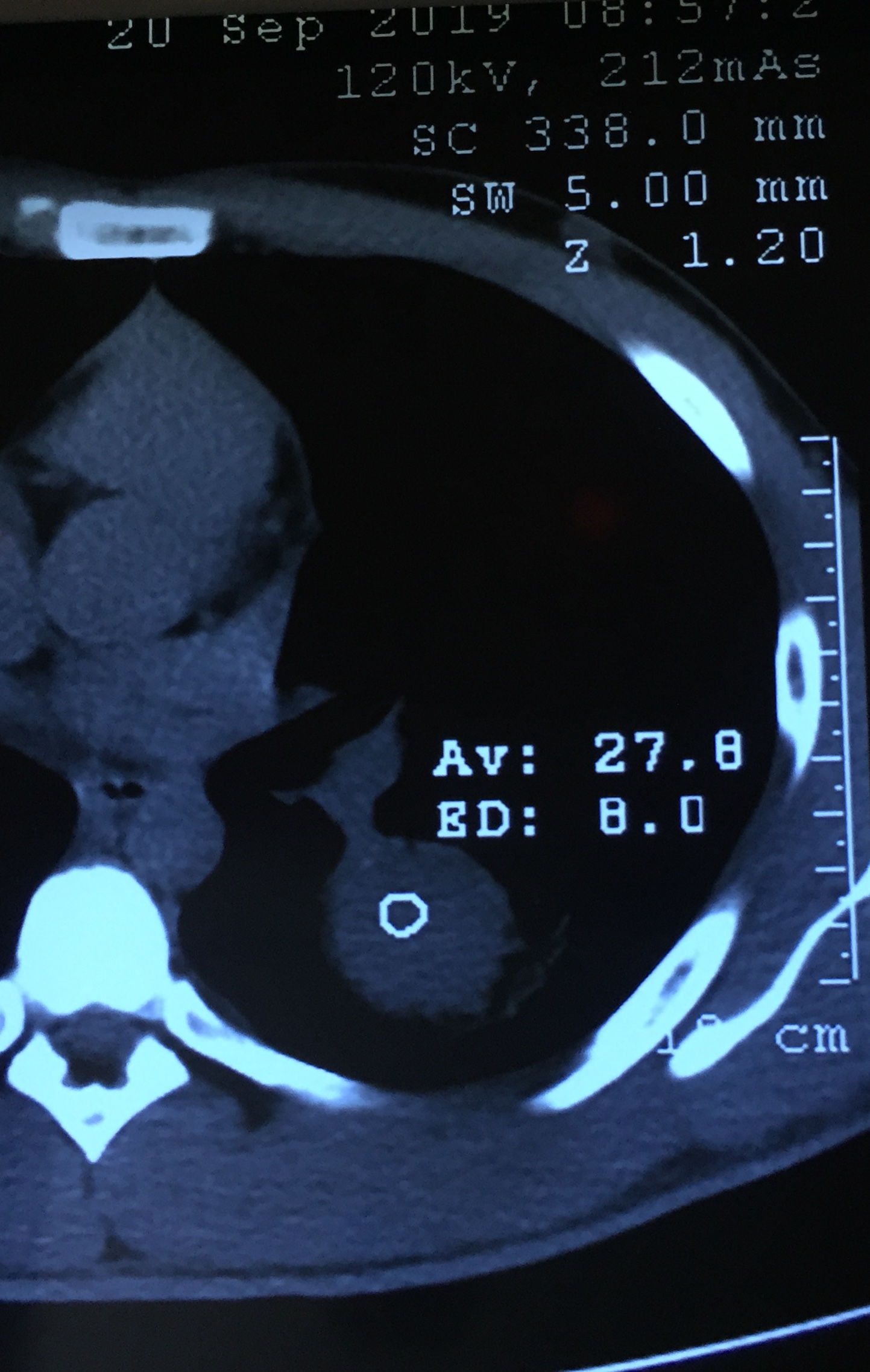

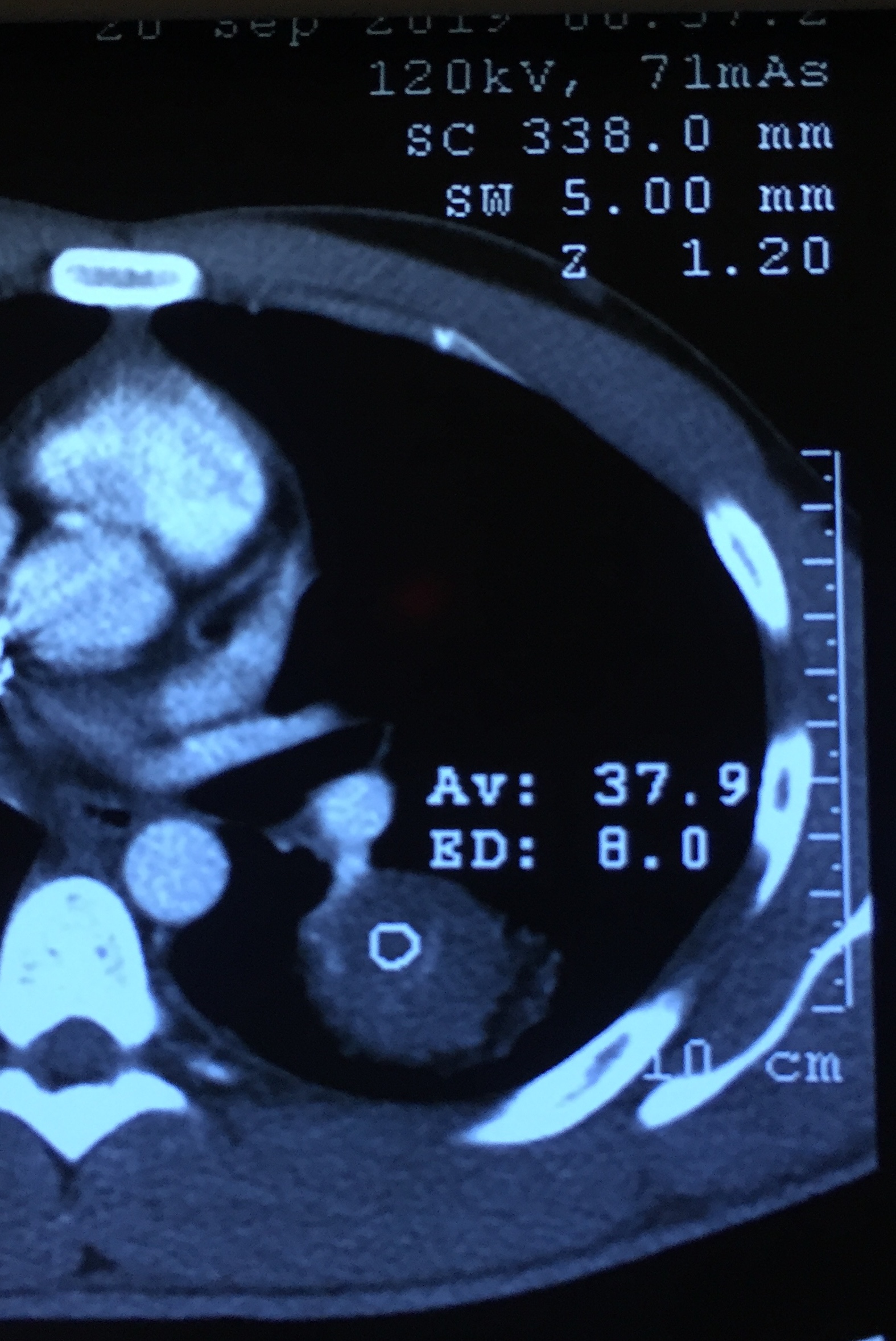

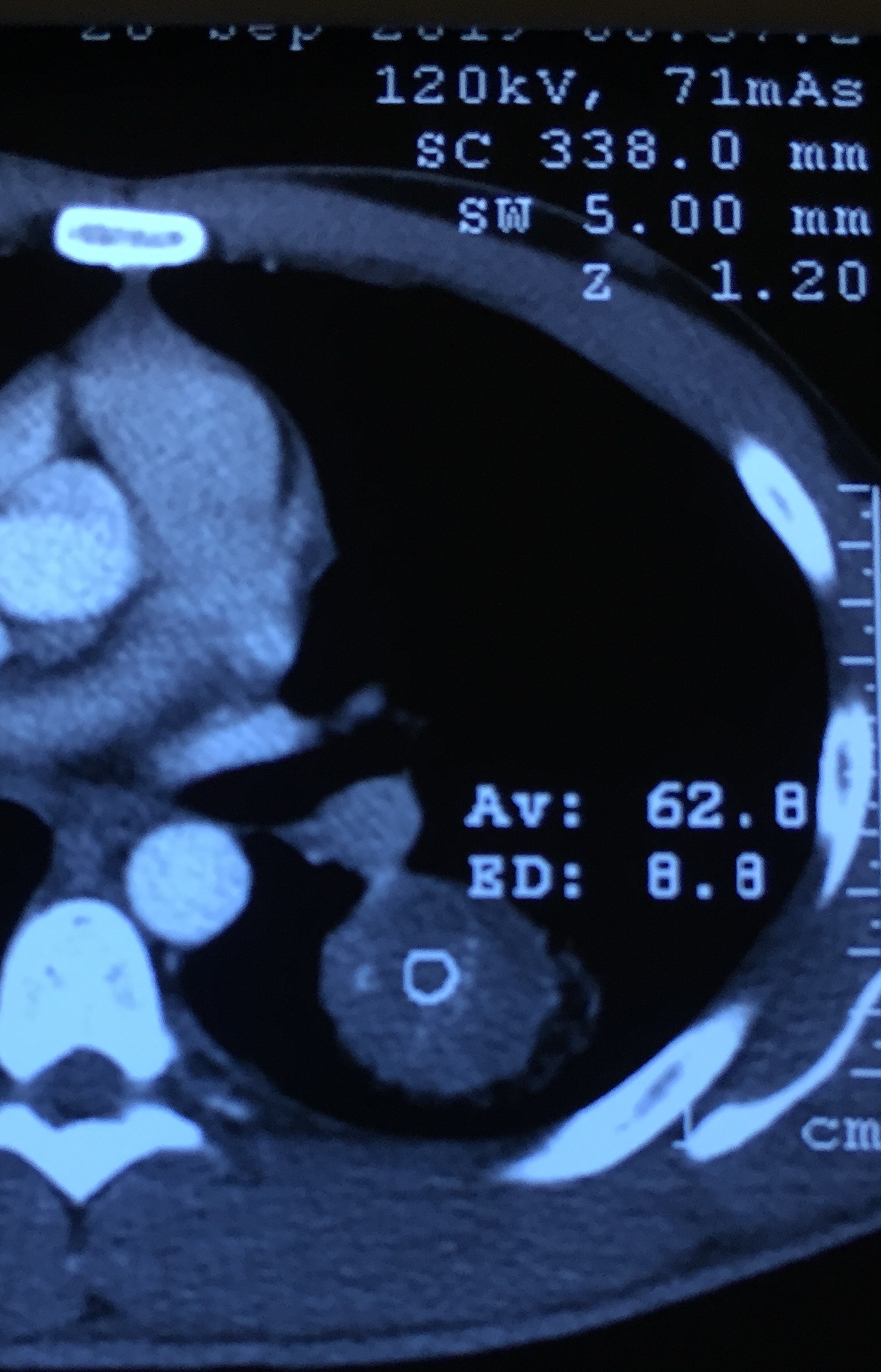

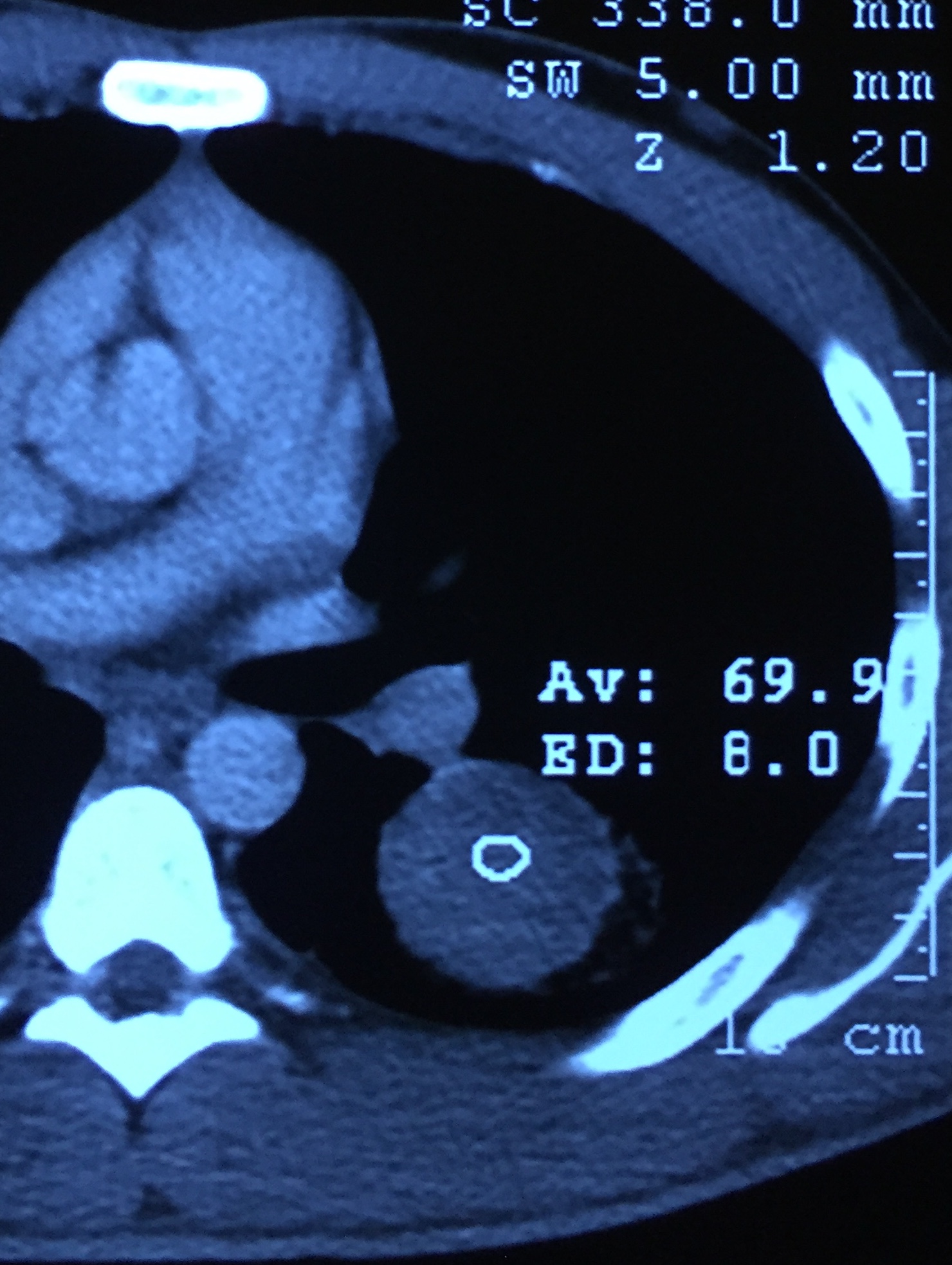

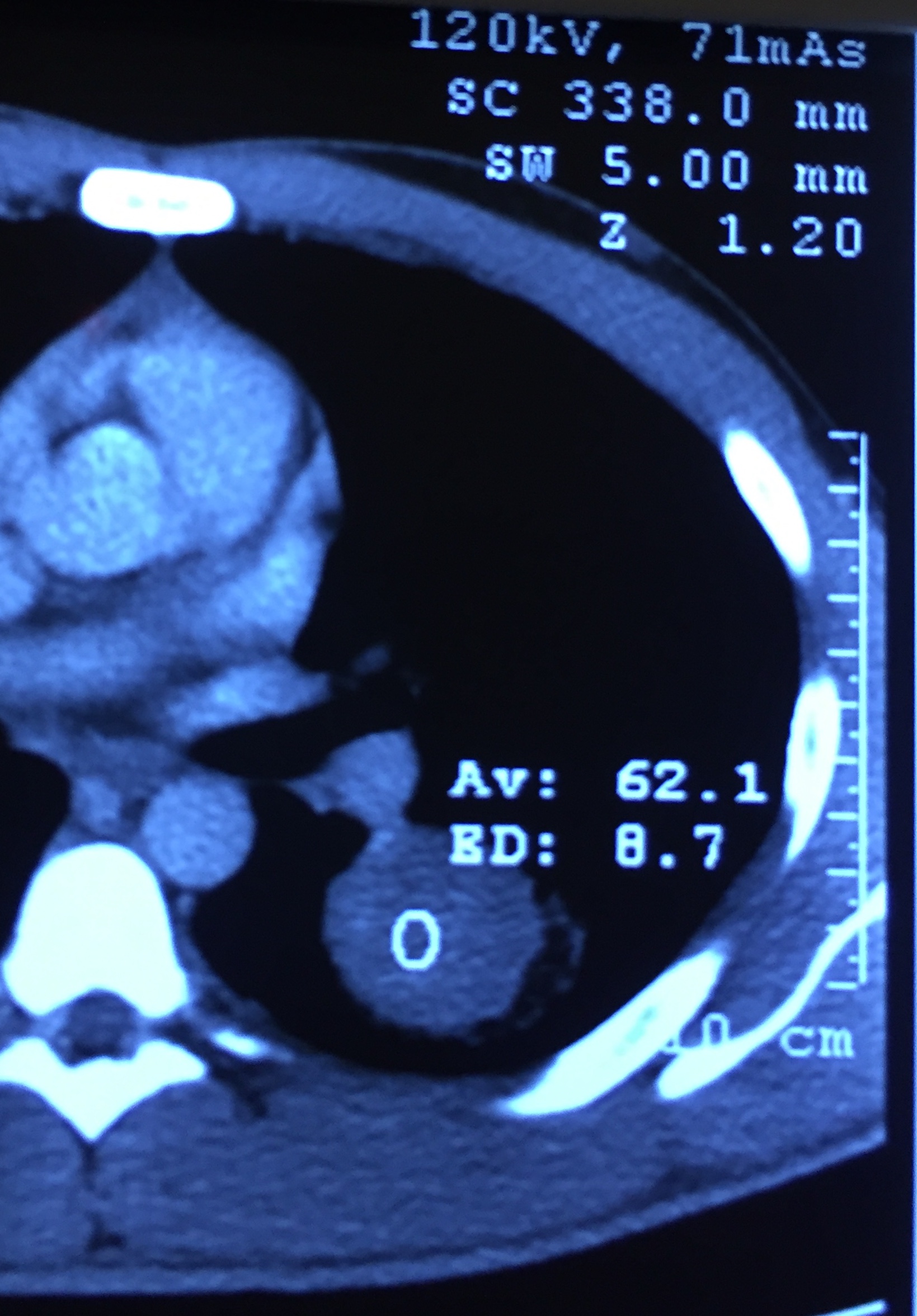


0s

60s

40s

20s

80s


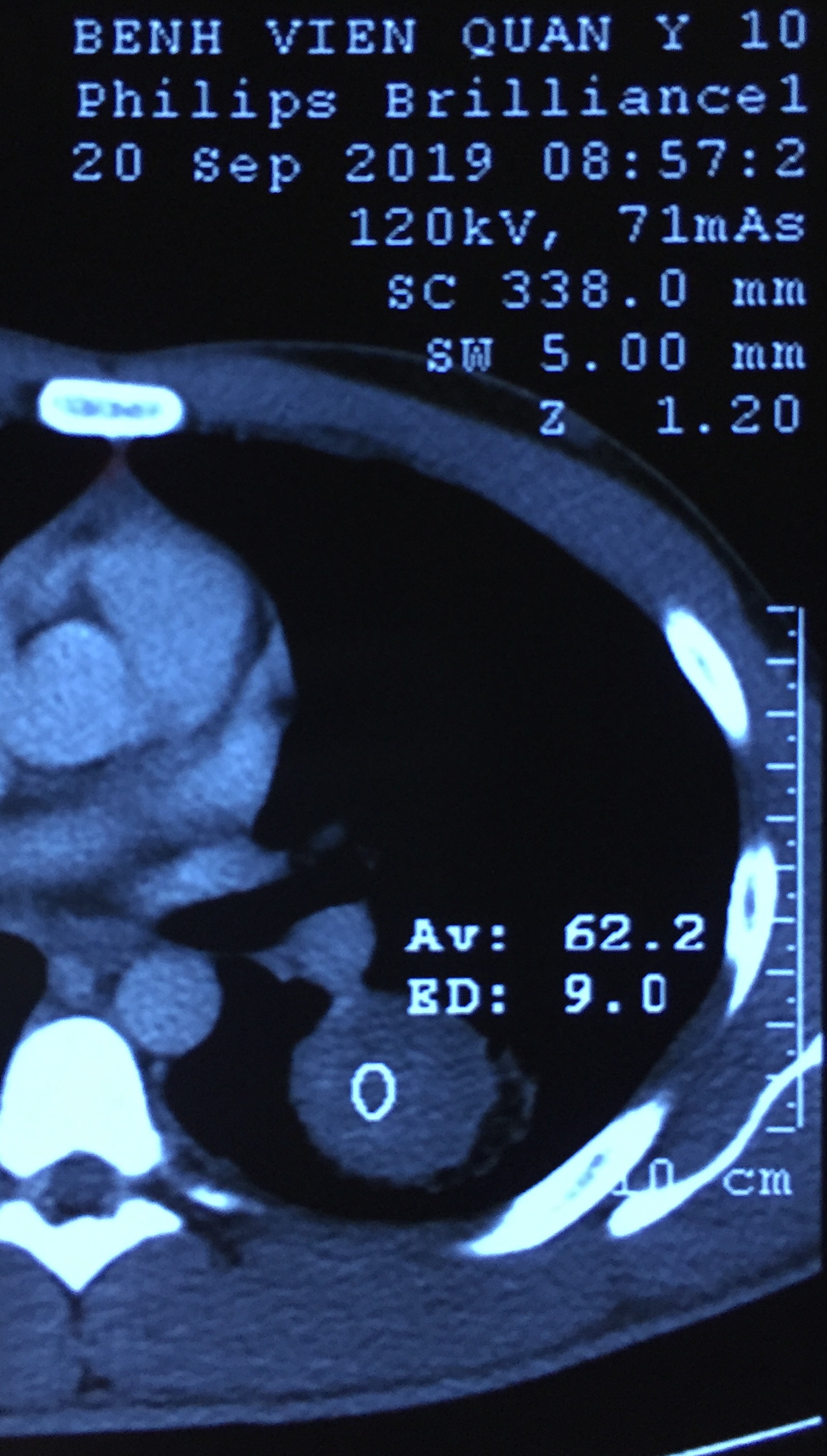

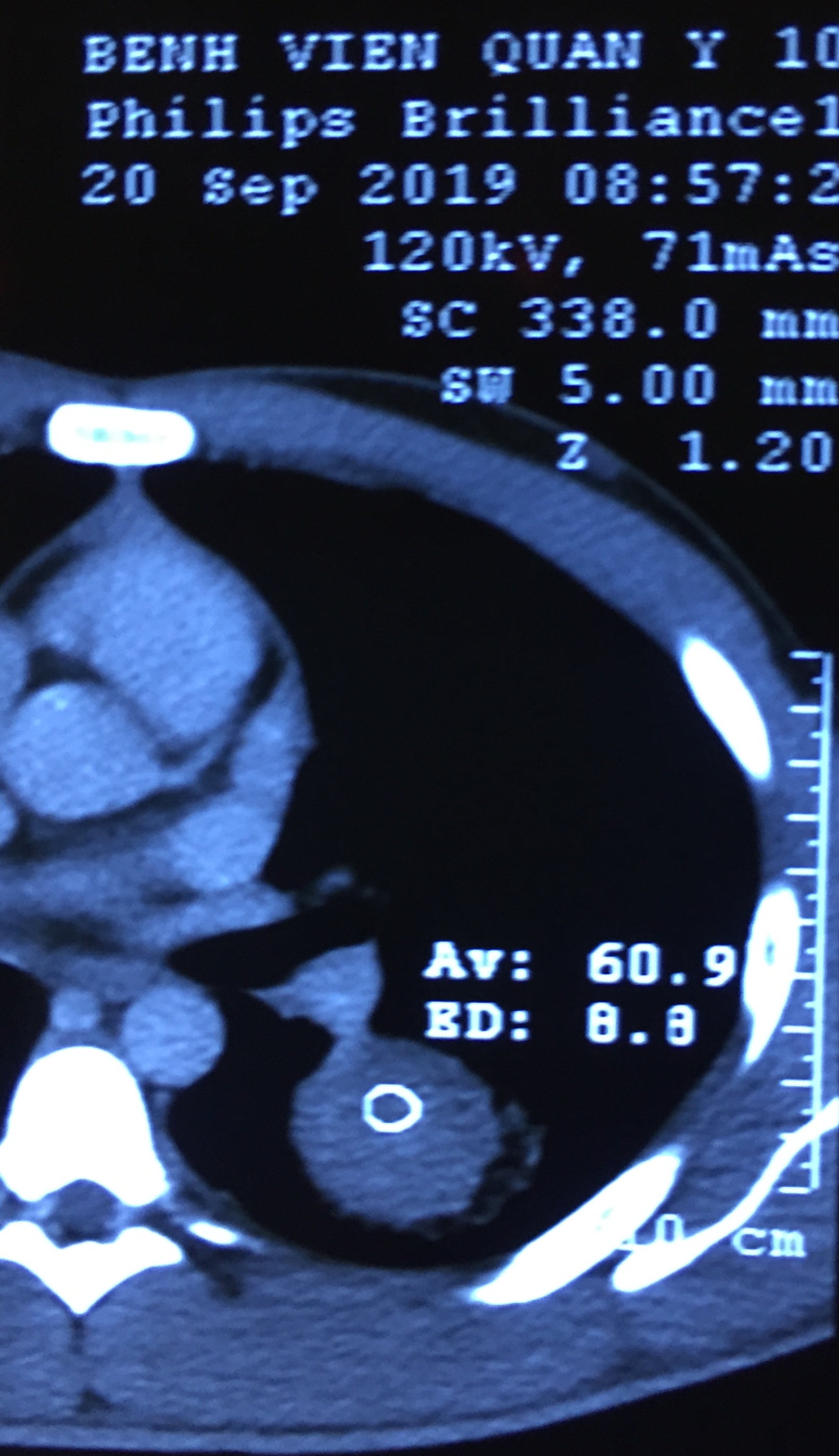

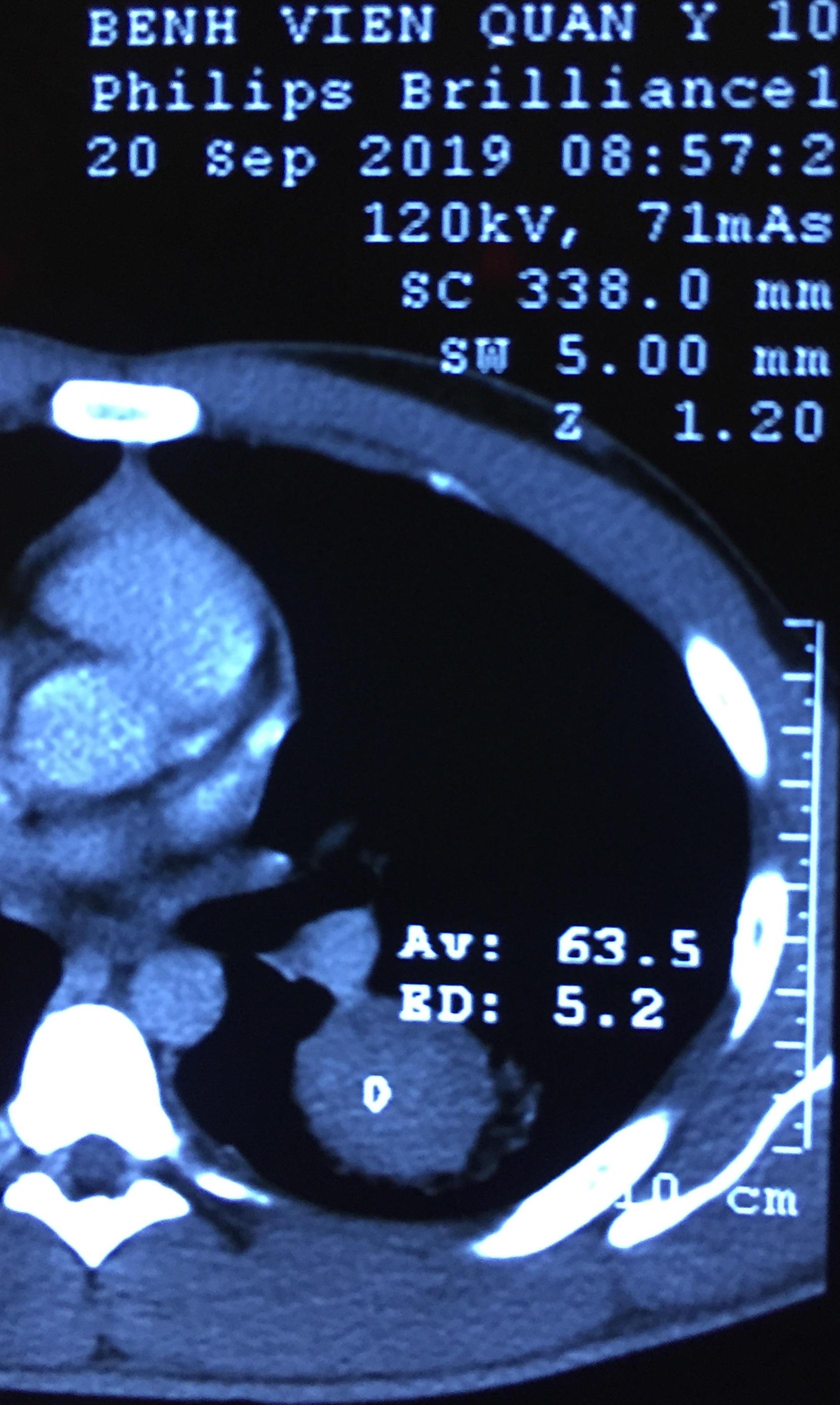

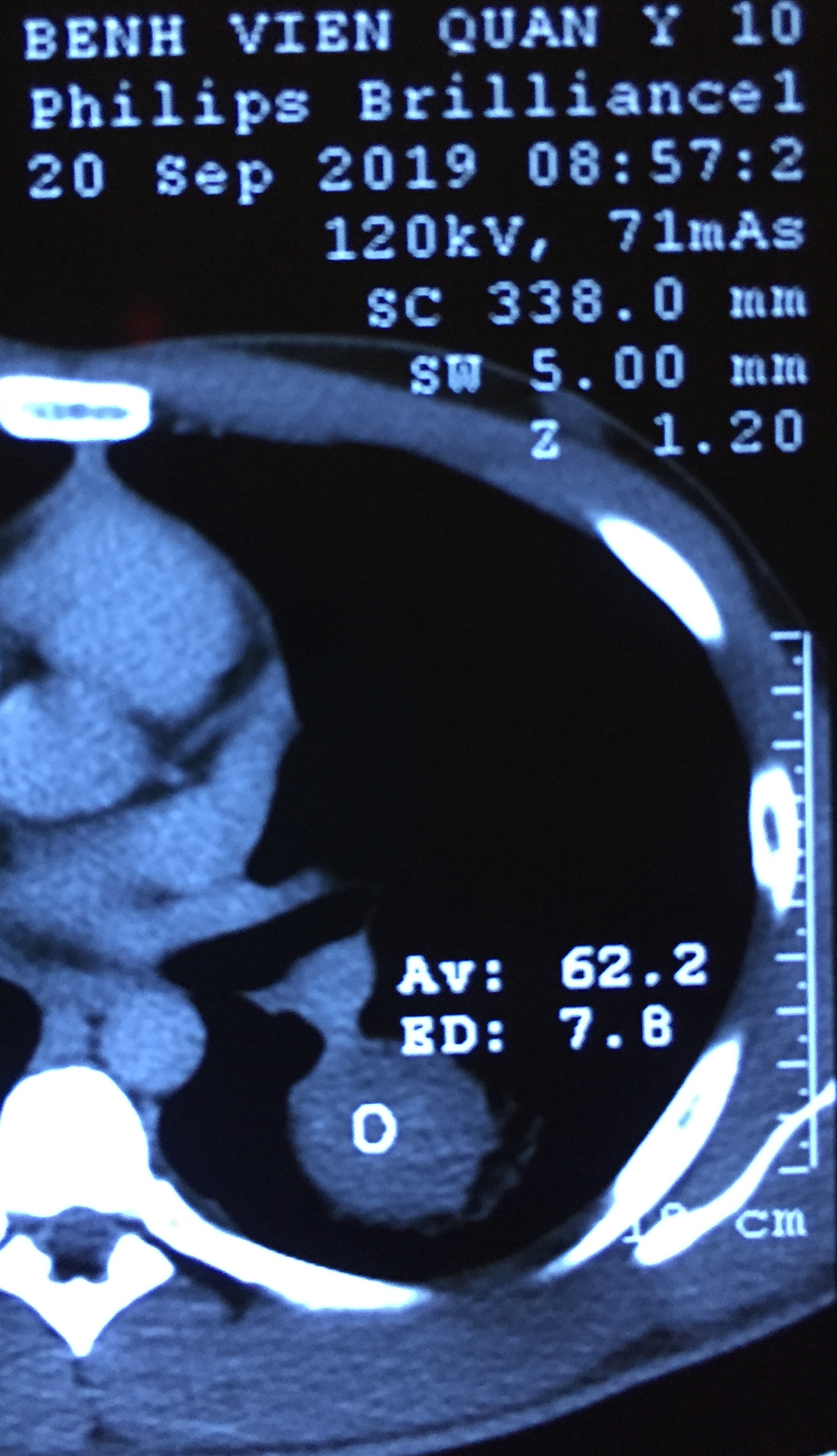

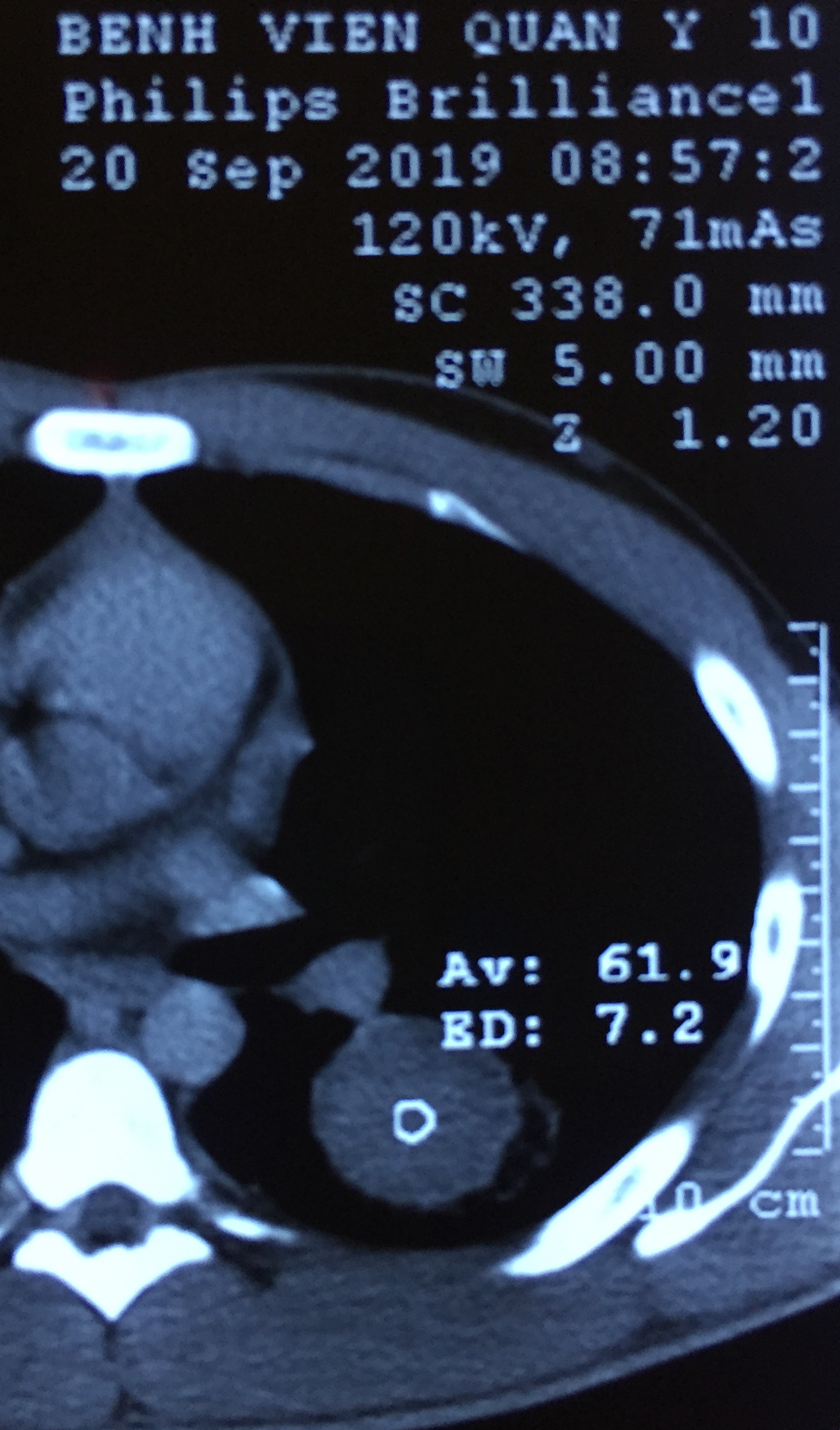


100s

120s

140s

160s

180s

*Time-attenuation curve obtained at 20-second intervals and at similar levels show enhancement dynamics of the nodule. Peak enhancement is 69.9 HU; net enhancement, 42.1 HU; and time to peak enhancement, 60 seconds.*

**Attenuation (HU)**
